# Childhood trauma and adulthood inflammation: a meta-analysis of peripheral C-reactive protein, interleukin-6 and tumour necrosis factor-α

**DOI:** 10.1038/mp.2015.67

**Published:** 2015-06-02

**Authors:** D Baumeister, R Akhtar, S Ciufolini, C M Pariante, V Mondelli

**Affiliations:** 1 King's College London, Department of Psychological Medicine, Institute of Psychiatry, Psychology and Neuroscience, London, UK; 2 King's College London, Department of Psychology, Institute of Psychiatry, Psychology and Neuroscience, London, UK; 3 Department of Psychology, University College London, London, UK; 4 King's College London, Department of Psychosis Studies, Institute of Psychiatry, Psychology and Neuroscience, London, UK; 5 National Institute for Health Research (NIHR) Mental Health Biomedical Research Centre, South London and Maudsley, NHS Foundation Trust and King's College London, London, UK

## Abstract

Childhood trauma confers higher risk of adulthood physical and mental illness; however, the biological mechanism mediating this association remains largely unknown. Recent research has suggested dysregulation of the immune system as a possible biological mediator. The present paper conducted a meta-analysis to establish whether early-life adversity contributes to potentially pathogenic pro-inflammatory phenotypes in adult individuals. A systematic search of Pubmed, PsycINFO, EMBASE, Scopus and Medline identified 25 articles for the meta-analysis, including 18 studies encompassing a sample of 16 870 individuals for C-reactive protein (CRP), 15 studies including 3751 individuals for interleukin-6 (IL-6) and 10 studies including 881 individuals for tumour necrosis factor-α (TNF-α). Random-effects meta-analysis showed that individuals exposed to childhood trauma had significantly elevated baseline peripheral levels of CRP (Fisher's *z*=0.10, 95% confidence interval (CI)=0.05–0.14), IL-6 (*z*=0.08, 95% CI=0.03–0.14) and TNF-α (*z*=0.23, 95% CI=0.14–0.32). Subgroup analyses for specific types of trauma (sexual, physical or emotional abuse) revealed that these impact differentially the single inflammatory markers. Moreover, meta-regression revealed greater effect sizes in clinical samples for the association between childhood trauma and CRP but not for IL-6 or TNF-α. Age, body mass index (BMI) and gender had no moderating effects. The analysis demonstrates that childhood trauma contributes to a pro-inflammatory state in adulthood, with specific inflammatory profiles depending on the specific type of trauma.

## Introduction

A large body of studies has so far supported the notion that childhood traumatic experiences, including physical, sexual and emotional abuse, neglect and separation from caregivers, significantly increase the risk of developing mental and physical illnesses later on in life,^1, 2^ but the biological mechanisms mediating this association remain unclear. More specifically, childhood trauma has been suggested to increase vulnerability to several psychiatric disorders, including depression,^3^ anxiety,^4^ psychosis^5^ and post-traumatic stress disorder,^4^ as well as several chronic physical health problems, including rheumatoid arthritis, cardiovascular disease, lung disease, metabolic syndrome and cancer.^6, 2, 7^ Furthermore, childhood trauma is associated with more unfavourable psychiatric outcomes, such as more recurrent and treatment-resistant depressive disorder^8^ and greater risk of suicidal behaviours.^9^ Studies in the recent decade have implicated the innate immune system in the relationship between childhood trauma and adulthood disease.

The primary purpose of the innate immune system is to provide an initial line of defence against pathogens as well as to contribute to the adaptive induction of sickness behaviour, a constellation of behavioural changes that facilitate recovery from infection while affecting mood and cognitive function. In humans, these symptoms are exemplified by those experienced by individuals who take pro-inflammatory cytokines, such as interferon-α, for medical indications, and include depression, anxiety, lethargy, fatigue, fragmented sleep, decreased appetite, psychomotor retardation and cognitive impairment.^10, 11^ Interestingly, elevated levels of inflammatory markers have been increasingly reported in psychiatric disorders^10^ as well as in individuals with a history of childhood trauma (see below). Of note, inflammatory signalling pathways are also known to impact on a network of biological systems extensively implicated in depression, including neuroendocrine, monoaminergic, oxidative, nitrosative and neurotrophic pathways.^12, 10^ In particular, the hypothalamic-pituitary-adrenal (HPA) is frequently dysregulated in physical and mental illnesses, and its altered function has been involved in the development of specific behavioural phenotypes associated with depression, such as early awakening and changes in weight and appetite.^13^ The HPA axis is also a powerful modulator of inflammatory activity and is in turn modulated by inflammatory processes,^14, 15, 16^ as well as being highly responsive to environmental adversities both in childhood and in adulthood.^13^ Taken together, these lines of evidence point to the activation of the immune system as one of the biological mechanisms underlying the pathogenesis of mental illness, especially in the context of early-life stress.

Several previous studies have reported an association between childhood trauma and increased levels of pro-inflammatory markers, most notably of the acute phase protein C-reactive protein (CRP), and of the cytokines interleukin-6 (IL-6) and tumour necrosis factor-α (TNF-α).^17^ However, in light of several non-significant findings as well as a significant amount of heterogeneity in methods, such as in the definition and assessment of childhood trauma, in the sample compositions and in the statistical approaches,^17^ a meta-analysis of the subject is warranted. Furthermore, whether any immune abnormalities are specific to one or more types of childhood trauma remains unclear.

The present meta-analysis aims to test whether childhood trauma is consistently associated with dysregulation of the inflammatory system in adulthood, thus increasing the vulnerability to health problems in adulthood. Moreover, we assess potential moderating factors in the association between childhood trauma and adulthood inflammation, including compositions of the samples and types of childhood trauma. We focus on studies examining peripheral levels of three key inflammatory markers, CRP, IL-6 and TNF-α, as these are the ones which have received most attention within the childhood trauma literature^17^ and therefore there is a sufficient number of studies to conduct a meta-analysis; these are also the inflammatory markers most frequently examined in psychiatric research.^10^

## Materials and methods

### Search strategy and selection

**Figure 1:**
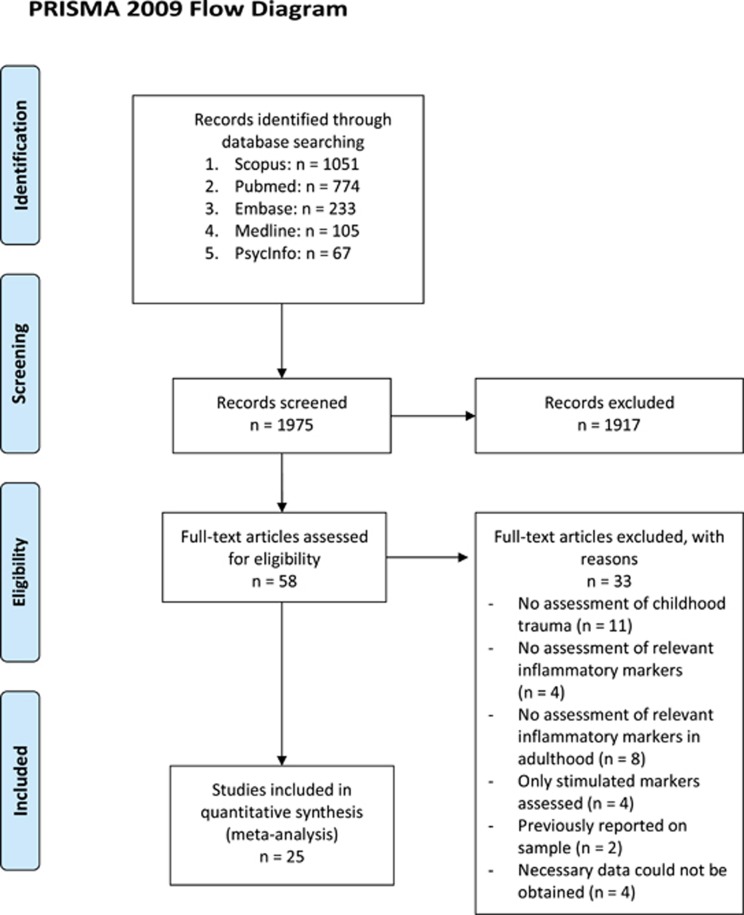
PRISMA diagram of the literature search.

A systematic review of the literature was performed using Pubmed, PsycINFO, EMBASE, Scopus and Medline for the subject headings ‘Childhood Maltreatment‘, ‘Childhood Trauma‘, ‘Childhood Adversity‘, ‘Early Life Stress‘, ‘Child Abuse‘ and ‘Child Neglect‘ cross-referenced separately with the terms ‘C-reactive Protein‘, ‘CRP‘, ‘Tumour Necrosis Factor‘, ‘TNF-α‘, ‘Cytokine‘, ‘Interleukin‘, ‘IL-6‘, ‘Inflammatory‘ and ‘Inflammation‘. The literature review was initially performed between 1 January and 31 March 2014, and updated on 15 February 2015. Articles were limited to research in human participants, published in English language. The initial search produced 1051 results on Scopus, 774 on Pubmed, 67 on PsycInfo, 233 on Embase and 105 on Medline. Articles were included if they provided original data about the association of any trauma experienced before age 18 (not including adverse socioeconomic status in childhood) with CRP, IL-6 and/or TNF-α levels in adulthood (individuals aged 18 or older). Titles and abstracts were scrutinised for appropriateness to the present objective. Sixty appropriate articles were identified for full-text analysis, of which 25 met criteria for inclusion in the present meta-analysis,^18, 19, 20, 21, 22, 23, 24, 25, 26, 27, 28, 29, 30, 31, 32, 33, 34, 35, 36, 37, 38, 39, 40, 41, 42^ including 18 for CRP, 15 for IL-6 and 10 for TNF-α (see Table 1), encompassing a sample of 16 870 participants for CRP, 3751 for IL-6 and 881 for TNF-α (see Figure 1).

### Data extraction and statistical analysis

Two authors (DB and RA) independently extracted data from eligible studies, and inconsistencies were resolved through discussion and consultation with other authors of the paper until consensus was reached. All studies were scored on the Selection Bias subscale of the Quality Assessment Tool;^43^ modified versions of this scale have previously been used in similar research.^44^ When data were not available, authors were contacted. All effect sizes were converted to Fisher's *z* before being entered into the analysis, to reflect the continuous nature of both levels of inflammatory markers as well as potential degrees of severity of childhood traumatic experiences, while decreasing the risk of bias associated with Pearson's *r*. As papers often normalise distribution of skewed biomarker data by utilising log-transformation, any raw data were transformed to logarithmic equivalents as described by Higgins *et al.*^45^

Since we hypothesise that the true effect sizes would differ depending on sample/exposure variations acting as moderating variables, random-effect models were chosen for the meta-analyses of main effects as well as meta-regressions and subgroup analyses. Samples were characterised for meta-regressions by the proportion of clinical participants (or those assessed for meeting criteria for clinical disorder), grouped into ‘any clinical disorders‘ (including physical and mental illnesses), ‘any psychiatric disorders‘ and ‘depressive disorder‘. Mean age, body mass index (BMI) and proportion of female participants were recorded for meta-regression. Statistical procedures were carried out using *Stata*,^46^ using the *metan* package for meta-analyses, the *metareg* package for meta-regressions and the *metafunnel* and *metabias* packages for assessment of publication bias. *P*-values below 0.05 were accepted as being statistically significant, and values below 0.1 were reported as trends.

## Results

### Main association of childhood trauma with inflammatory markers

**Figure 2:**
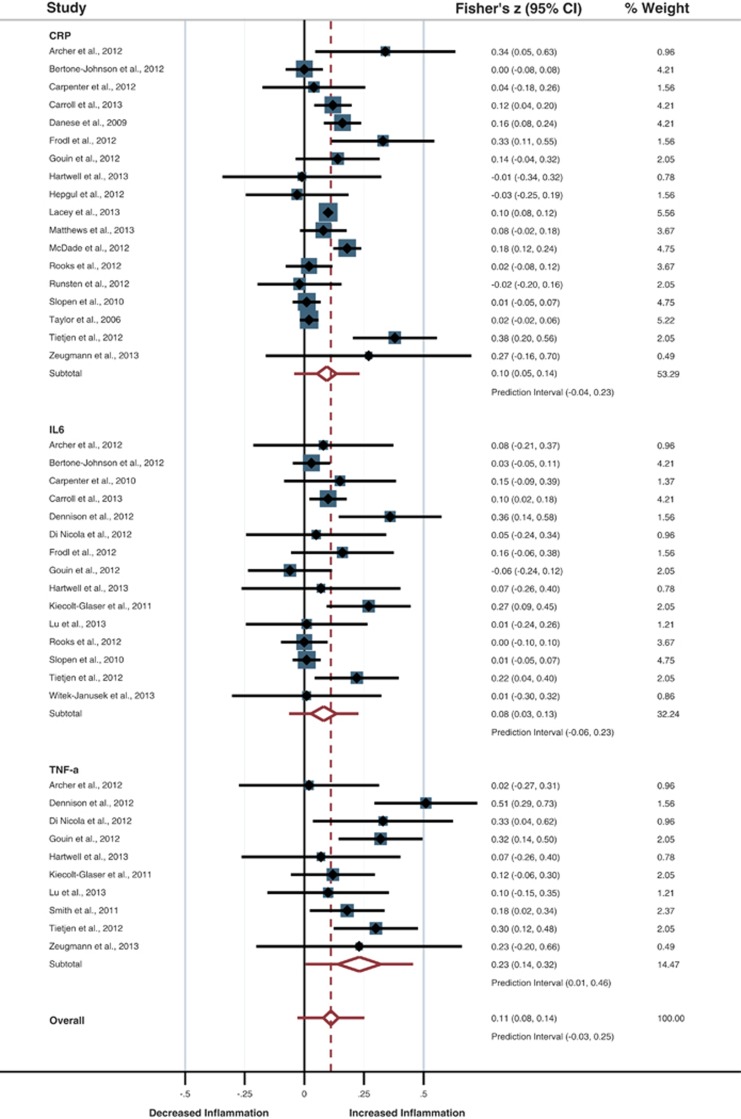
Forest plot presenting the main association of childhood trauma with inflammatory markers.

Meta-analysis for the main effects showed consistently elevated levels of inflammation associated with childhood trauma, with small effect sizes, for CRP (Fisher's *z*=0.10, df=17, *P*<0.001, 95% confidence interval (CI)=0.05–0.14, prediction interval (PI)=−0.04–0.23), IL-6 (*z*=0.08, df=14, *P*=0.003, 95% CI=0.03–0.14, PI=−0.06–0.23) and TNF-α (*z*=0.23, df=9, *P*<0.001, 95% CI=0.14–0.32, PI=0.01–0.46; see Figure 2). When grouped into one inflammatory factor, effect sizes were small yet significant (*z*=0.11, df=42, *P*<0.001, 95% CI=0.08–0.14, PI=−0.03–0.25). High heterogeneity was observed for CRP (*P*<0.001, *I*^2^=72.7%, *τ*^2^=0.004), while low to moderate heterogeneity scores were observed for IL-6 (*P*=0.03, *I*^2^=44.5%, *τ*^2^=0.004) and TNF-α (*P*=0.11, *I*^2^=36.8%, *τ*^2^=0.007), and moderate to high heterogeneity was observed for the overall inflammatory factor (*P*<0.001, *I*^2^=65.6%, *τ*^2^=0.005).

### Type of trauma

**Figure 3:**
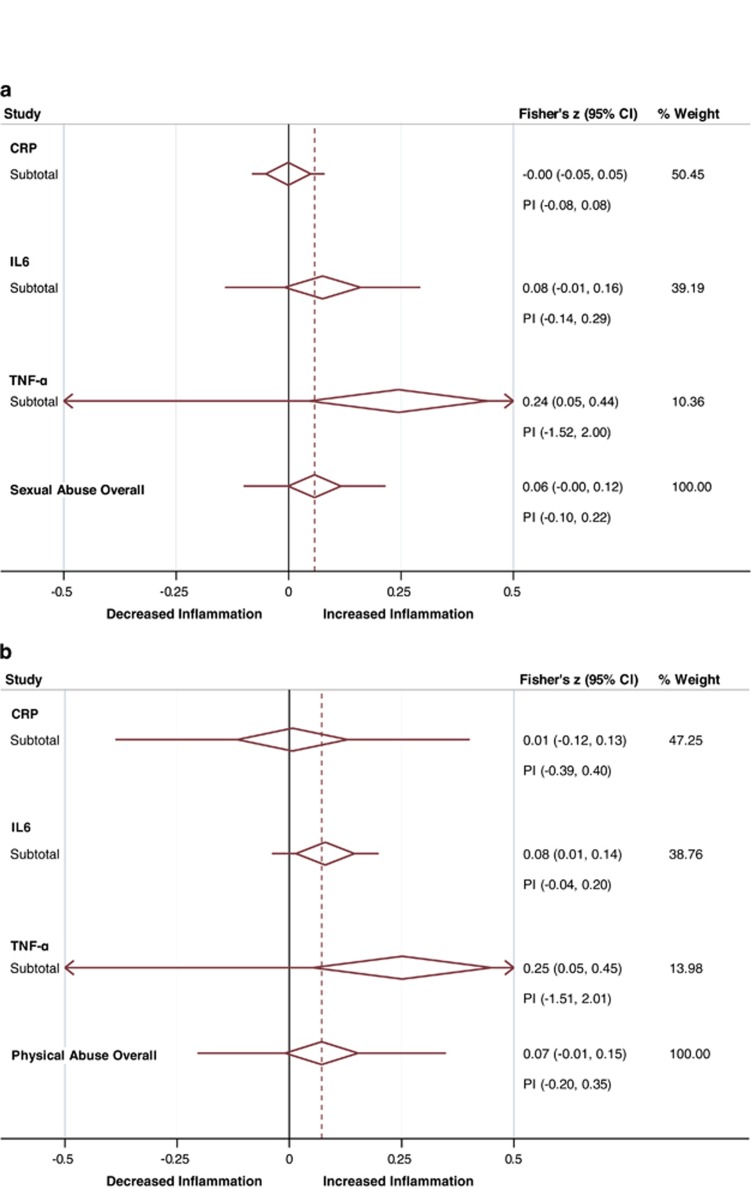
Collapsed forest plots presenting the association of sexual (**a**) and physical (**b**) abuse with inflammatory markers.

Sub-group analyses for childhood sexual abuse (Figure 3a) showed this trauma type to be significantly associated with a small effect size for TNF-α (*z*=0.24, df=2, *P*=0.02, 95% CI=0.05–0.44) and a trend toward a small effect size for IL-6 (*z*=0.08, df=4, *P*=0.08, 95%CI=−0.01–0.16), while no such results were found for CRP (*z*=−0.001, df=4, *P*=0.98, 95% CI=−0.05–0.05). Heterogeneity scores were not significant for estimates of TNF-α (*P*=0.25, *I*^2^=28.5%, *τ*^2^=0.009), IL-6 (*P*=0.21, *I*^2^=32.4%, *τ*^2^=0.003), or CRP (*P*=0.62, *I*^2^=0.0%, *τ*^2^=0.0).

Sub-group analyses for childhood physical abuse (Figure 3b) similarly showed significant associations with small effect sizes for TNF-α (*z*=0.25, df=2, *P*=0.01, 95% CI=0.05–0.45) and IL-6 (*z*=0.08, df=5, *P*=0.02, 95%CI=0.02–0.15), but not for CRP (*z*=0.007, df=5, *P*=0.91, 95% CI=−0.12–0.13). Heterogeneity scores were again not significant for estimates of TNF-α (*P*=0.25, *I*^2^=28.5%, *τ*^2^=0.009), IL-6 (*P*=0.35, *I*^2^=9.8%, *τ*^2^=0.007), but significant for CRP (*P*<0.001, *I*^2^=79.3%, *τ*^2^=0.016).

Since only a small number of studies investigated emotional abuse, meta-analysis for this trauma was only possible for CRP and IL-6, but not for TNF-α. Neither CRP (*z*=0.03, df=3, *P*=0.31, 95% CI=−0.03–0.10) nor IL-6 (*z*=−0.008, df=2, *P*=0.87, 95% CI=−0.10–0.08) was significantly associated with childhood emotional abuse. Heterogeneity did not reach significance for CRP (*P*=0.81, *I*^2^=0.0%, *τ*^2^=0.0) or IL-6 (*P*=0.65, *I*^2^=0.0%, *τ*^2^=0.0).

Sub-group analyses for parental absence during childhood was only possible for CRP, revealing a significant association with a small effect size (*z*=0.11, df=2, *P*=0.001, 95% CI=0.02–0.19) and significant heterogeneity (*P*=0.006, *I*^2^=80.4%, *τ*^2^=0.004).

### Moderating effects of sample populations

To investigate whether clinical samples accounted for heterogeneity in findings, meta-regressions were carried out for the proportion of participants with ‘any clinical disorders' (including physical and mental illnesses), ‘psychiatric disorders' and ‘depressive disorder'. Again, random-effect models were chosen as the most conservative model. While meta-regressions showed that samples including any clinical disorders showed augmented trend-level effect sizes for CRP (*t*=2.09, *P*=0.054, *I*^2^_res_=70.23%, *τ*^2^=0.005), explaining 9.81% of the between-sample variance, this was not significant for TNF-α (*t*=−0.07, *P*=0.95, *I*^2^_res_=43.8%, *τ*^2^=0.01) and IL-6 (*t*=0.48, *P*=0.64, *I*^2^_res_=42.89%, *τ*^2^=0.006).

The proportion of patients with any psychiatric disorders did not moderate the associations between childhood trauma and CRP (*t*=0.02, *P*=0.98, *I*^2^_res_=74.0%, *τ*^2^=0.007), IL-6 (*t*=0.23, *P*=0.82, *I*^2^_res_=48.5%, *τ*^2^=0.006) or TNF-α (*t*=0.54, *P*=0.61, *I*^2^_res_=42.7%, *τ*^2^=0.01). Similarly, the proportion of depressed patients did not moderate the association between childhood trauma with and CRP (*t*=1.07, *P*=0.30, *I*^2^_res_=73.5%, *τ*^2^=0.006), IL-6 (*t*=0.44, *P*=0.66, *I*^2^_res_=47.6%, *τ*^*2*^=0.006) or TNF-α (*t*=0.22, *P*=0.83, *I*^2^_res_=43.3%, *τ*^2^=0.01).

No significant results were found for moderating effects for age, BMI or gender, and there was no evidence of publication bias, although significant and trend-level moderating effects of selection bias were found for IL-6 and CRP, respectively. For additional information on sensitivity analyses, please see the Supplementary Material.

## Discussion

### Main findings and proposed mechanism

The present meta-analysis finds a significant association between childhood trauma and the inflammatory markers, with effect sizes being greatest for TNF-α (*z*=0.20, 95% CI=0.10–0.29), followed by IL-6 (*z*=0.09, 95% CI=0.04–0.15) and then CRP (*z*=0.08, 95% CI=0.04–0.11). As such, this provides strong evidence that childhood traumatic events significantly impact on the inflammatory immune system, with trajectories reaching into adulthood, thus offering a potential molecular pathway by which early trauma confers vulnerability to developing psychiatric and physical disorders later in life.

The molecular mechanisms that account for these long-term changes in immune function need to be further explored. Putatively, changes in epigenetic regulation of gene expression may be responsible for this increased immune activation; this appears plausible in view of the considerable evidence that childhood trauma induces modifications of HPA- and neuroplasticity-related methylation patterns.^47, 48, 49^ In particular, early trauma leads to greater methylation of the glucocorticoid receptor (GR) and greater demethylation of FKBP5.^48, 50, 51, 52^ The increased methylation of the GR correlates with reduced GR function as shown by impaired negative feedback of the HPA axis,^52^ while FKBP5 is a heat-shock protein that binds and thereby inhibits the cytosolic GR. As the GR itself is a crucial regulator of inflammatory activity, lower expression and function of the GR due to epigenetic suppression may allow for this exacerbated inflammatory activity. Notably, increased inflammation itself can then maintain and exacerbate the impaired GR function,^14, 15, 16^ thus leading to sustained GR resistance into adulthood. Notably, based on differences in effects sizes found in the present analysis, the association between childhood trauma and adulthood inflammation is stronger for inflammatory pathways related to TNF-α and, indeed, the GR is crucial in regulating TNF-α signalling and TNF-induced cytokine production, as well as conveying protection against TNF-related tissue damage.^53^

The present paper also found evidence that individual types of trauma exposure impact differentially on the inflammatory markers: most interestingly, physical and sexual abuse is associated with significant increased TNF-α and IL-6, but not CRP. Conversely, CRP seemed to be primarily related to parental absence during early development. Interestingly, rodent models have demonstrated that maternal separation is associated with elevated TNF-α levels in the periphery and cerebrospinal fluid^54^ as well as in prefrontal and hippocampal brain regions.^55^ These results stress the need for assessing the potential different effects of each type of trauma in future research. Moreover, this finding raises the question as to why different types of childhood trauma are associated with different aspects of inflammatory dysregulation.

While there is currently no clear answer to this question, several variables associated with individual trauma types, including chronicity and context of the stressor, age of exposure, duration of exposure or relationship to the perpetrator, may offer some insight. For example, it has been shown in adolescent women that episodic stress in the context of high chronic stress leads to reduced GR expression, while GR expression is increased in the absence of chronic stress.^56^ Moreover, there is evidence that different types of trauma impact differentially on mental health: specific subtypes of anxiety disorders appear to develop depending on whether one is exposed to physical or sexual childhood abuse,^4^ and childhood sexual abuse is particularly associated with the development of auditory verbal hallucinations in psychosis.^5^ The effect of early trauma on behaviour in adulthood may be further modulated by other developmental insults: Giovanoli *et al.*^57^ showed that prenatal immune viral challenge in mice synergistically interacts with peripubertal stress exposure thus increasing the vulnerability to develop neuropathological behaviours later on in life. Interestingly, these mice show transient neuroimmunological changes, evident during adolescence but not in adulthood, suggesting the presence of sensitive periods of peripubertal brain maturation which might differentially influence behavioural outcomes. Further supporting the role of immune-related molecular pathways in the long-term consequences of early-life trauma, previous preclinical studies have shown that early-life stress tends to have a programming effect on neuroimmune functions, leading to a pro-inflammatory state in adulthood, which in turn can trigger an exaggerated cytokine secretion and increase in microglia activity following an immune challenge. In particular, maternal deprivation early in life has been shown to enhance IL-1β responsiveness in adulthood, due to elevated IL-1 receptor levels at the post-synapse of adult hippocampal neurons.^58^ Moreover, prenatal stress in mice increases expression of IL-1β and TNF-α in the hippocampus during adulthood, and this pro-inflammatory state results in an enhanced activation of microglia and astrocytes in response to an immune challenge.^59^

In the case of CRP, the impact of childhood trauma on adulthood inflammation may be characterised by synergistic effects with the presence of ‘state‘ (current) ill health, as the effect sizes were significantly greater in clinical samples, including patients with cancer. However, this effect was not observed when focussing exclusively on psychiatric disorders, suggesting that in these individuals the ‘trait‘ effects (the history of childhood trauma) may be the main driver of the immune activation. It should be noted that, although the increase in inflammatory activity is not comparable to that of acute systemic inflammatory disorders, it still has clinical relevance. We know that 95% of CRP values in healthy individuals range between 0.07 and 5.25 mg l^−^^1^,^60^ which translates into a mean of 2.66 mg l^−1^ and a standard deviation of 4.18 mg l^−1^; this is in line with epidemiological findings, showing mean CRP values in the general population ranging between 1.4 and 2.9 mg l^−1^.^61^ Considering the standardised mean difference of 0.2 (equivalent to a Fisher's *z* of 0.1), individuals with a history of childhood trauma would show an average CRP increase of 0.84 mg l^−1^, or a mean CRP value of 3.5 mg l^−1^, which is above the threshold of 3 mg l^−1^ acknowledged as risk factor for future heart attack, stroke and development of diabetes.^62^ Thus, inflammatory activation as a consequence of childhood trauma is best conceptualised as a subtle effect that is likely to have a significant impact on physical and mental health.

### Limitations

The present meta-analysis found significant yet small effect sizes in the context of particularly high levels of heterogeneity, which remained after subsequent meta-regressions and subgroup analyses. However, this may reflect the fact that the papers reviewed here show great variations in both theoretical and methodological approaches, and that there is considerable variation in the assessment of inflammatory markers (see Table 1). Such variations in measurement instruments may be associated with differences in sensitivity, which, especially in the context of the apparently small changes associated with childhood trauma, may create type II errors. Sensitivity analysis looking at the different instruments to assess trauma shows larger effect sizes for validated instruments when compared with non-standardised assessments (see Supplementary material). Notably, sensitivity analysis also shows that prospective studies show greater effect sizes and lower heterogeneity than studies using retrospective trauma assessment (see Supplementary material). In light of these various potential sources of heterogeneity, the choice of random-effect models, albeit more conservative, was indeed the most appropriate for the present purpose. While the lack of evidence for publication bias suggests that the present findings are not an artefact of a distorted literature, the potential impact of selection biases cannot be ruled out given its effect in the results for IL-6 and CRP. Finally, not all the studies included in the meta-analysis explicitly reported acute infection as exclusion criteria for their analyses, and this could have partially affected the findings of those individual studies.

### Implications

The present meta-analysis demonstrates a significant association between childhood trauma and increased immune activation in adulthood, and highlights possible differential effects of different types of trauma as well as heterogeneity in the findings. Future research should further investigate the molecular mechanisms behind this association, and particularly whether inflammatory and neuroendocrine changes actually occur in parallel in the same individuals, and how these changes are embedded from a molecular point of view. Contextualising these data within a wider array of biological systems may be crucial in identifying why some individuals go on to develop physical or psychiatric disorders, whereas other remain resilient in the face of exposure to trauma.

These findings are also clinically relevant. Besides the potential impact of the increased inflammation on metabolic outcomes and physical illness, as discussed above, the assessment of inflammatory markers may also aid the development of prevention and treatment strategies. For example, a recent meta-analysis has demonstrated that elevations in CRP and IL-6 appear to precede the development of depressive disorders,^63^ and that patients with increased inflammation are less likely to respond to conventional antidepressants,^64^ and more likely to respond to adjunctive anti-inflammatory treatment.^65^ Thus, assessment of childhood trauma in conjunction with that of inflammatory markers may prove crucial in developing more effective prevention strategies and treatments, affecting long-term mental health outcomes.

## Figures and Tables

**Table 1:** Systematic overview of articles included in the meta-analysis

*Name*	*Sample size*^a^	*Inflammatory markers*	*Population*	*Mean age*	*Female proportion (%)*	*Design*	*Trauma measure*	*Assay*
Archer *et al.*^18^	46	CRP, IL-6, TNF-α	Clinical—Cancer patients	65.64	46.2	Retrospective	CTQ	Multi-spot protocol
Bertone-Johnson *et al.*^19^	702	CRP, IL-6	General population	42.67	100.0	Retrospective	n.s.	Immunonephelometry
								ELISA
Carpenter *et al.*^21^	69	IL-6	General population	26.80	60.9	Retrospective	CTQ	ELISA
Carpenter *et al.*^20, 52^	92	CRP	General population	30.50	51.1	Retrospective	CTQ	Immunonephelometry
Carroll *et al.*^40^	765	CRP, IL-6	General population	40.00	57.3	Retrospective	RFQ	Immunonephelometry
								ELISA
Danese *et al.*^39^	633	CRP	General population	32.00	48.0	Prospective	n.s.	Immunotubodimetry
Dennison *et al.*^22^	80	IL-6, TNF-α	Clinical—FEP patients vs HC	37.27	53.8	Retrospective	CTQ	ELISA
Di Nicola *et al.*^23^	48	IL-6, TNF-α	Clinical—FEP patients vs HC	27.35	35.4	Retrospective	CECA	ELISA
Frodl *et al.*^24^	83	CRP, IL-6	Clinical—MDD patients vs HC	39.12	59.0	Retrospective	CTQ	ELISA
Gouin *et al.*^25^	130	CRP, IL-6, TNF-α	General population	65.13	82.3	Retrospective	CTQ	High Sensitivity Immunoassay
								Chemiluminescence
Hartwell *et al.*^26^	38	CRP, IL-6, TNF-α	General population	35.69	51.3	Retrospective	ETI	Multiplex Bead Array
Hepgul *et al.*^27^	80	CRP	Clinical—FEP patients vs HC	26.69	34.4	Retrospective	CECA	High Sensitivity Immunoassay
Kiecolt-Glaser *et al.*^28^	132	IL-6, TNF-α	General population	69.69	72.0	Retrospective	CTQ	High Sensitivity Immunoassay
Lacey *et al.*^29^	7462	CRP	General population	42.00	49.5	Prospective	n.s.	Immunonephelometry
Lu *et al.*^30^	65	IL-6, TNF-α	Clinical—MDD patients vs HC	29.32	55.4	Retrospective	CTQ	Cytokine Antibody Array
Matthews *et al.*^40^	443	CRP	General population	45.70	100.0	Retrospective	CTQ	Immunonephelometry
McDade *et al.*^32^	1622	CRP	General population	20.90	NA	Prospective	n.s.	Immunoturbodimetry
Rooks *et al.*^41^	482	CRP, IL-6	General population	55.00	0.0	Retrospective	ETI	Chemiluminscence
								ELISA
Runsten *et al.*^33^	116	CRP	General population	42.89	100.0	Retrospective	RFQ	ELISA
Slopen *et al.*^34^	999	CRP, IL-6	General population	57.90	55.4	Retrospective	n.s.	Immunonephelometry
								ELISA
Smith *et al.*^35^	177	TNF-α	Clinical—PTSD patients vs HC	NA	NA	Retrospective	CTQ	ELISA
Taylor *et al.*^42^	3248	CRP	General population	40.10	54.7	Retrospective	RFQ	Immunonephelometry
Tietjen *et al.*^36^	141	CRP, IL-6, TNF-α	Clinical—Migraneurs	36.98	100.0	Retrospective	ACEQ	Immunonephelometry
			vs HC					Bead-based sandwich immunoassay
Witek Janusek *et al.*^37^	40	IL-6	Clinical—Breast cancer patients	55.60	100.0	Retrospective	CTQ	ELISA
Zeugmann *et al.*^38^	23	CRP, TNF-α	Clinical—MDD patients	47.80	68.0	Retrospective	CTQ	ELISA

Abbreviations: ACEQ, Adverse Childhood Experiences Questionnaire; CECA, childhood experiences of care and abuse; CRP, C-reactive protein; CTQ, Childhood Trauma Questionnaire; ELISA, enzyme-linked immunosorbent assay; ETI, Early Trauma Inventory; FEP, first episode psychosis; HC, healthy control; IL-6, interleukin 6; MDD, major depressive disorder; NA, not available; n.s., non-standardised; PTSD, post-traumatic stress disorder; RFQ, Risky Families Questionnaire; TNF-α, tumor necrosis factor-α.

a Sample sizes may vary depending on individual inflammatory markers.
