# Supplementary material for: Childhood trauma and adulthood inflammation: a meta-analysis of peripheral C-reactive protein, interleukin-6 and tumour necrosis factor-α
Source: Mol Psychiatry. 2015 Jun 2;21(5):642–9. doi: 10.1038/mp.2015.67 (PMC4564950; doi:10.1038/mp.2015.67)
Supplement: Supplementary Material [file mp201567x1.doc]

**Supplementary Material**

## Moderating effects of control variables and design

Meta-regression for the number of adjustments carried out showed no significant moderating effects on IL-6 (*t* = -1.44, *p* = 0.17, *I^2^_res_* = 41.7%, *τ^2^* = 0.005), CRP (*t* = -0.56, *p* = 0.58, *I^2^_res_* = 74.2%, *τ^2^* = 0.007) or TNF-α (*t* = 0.07, *p* = 0.95, *I^2^_res_* = 43.6%, *τ^2^* = 0.011). When individual meta-regressions were carried out, neither mean age, mean BMI nor gender proportion of study samples moderated the effects of childhood trauma with either CRP (Age: *t* = -0.83, *p* = 0.42, *I^2^_res_* = 67.0%, *τ^2^* = 0.005; BMI: *t* = -0.00, *p* = 0.10, *I^2^_res_* = 74.4%, *τ^2^* = 0.007; Gender: *t* = 0.56, *p* = 0.59, *I^2^_res_* = 71.1%, *τ^2^* = 0.007), IL-6 (Age: *t* = -0.93, *p* = 0.37, *I^2^_res_* = 40.5%, *τ^2^* = 0.005; BMI: *t* = -0.62, *p* = 0.55, *I^2^_res_* = 49.4%, *τ^2^* = 0.007; Gender: *t* = 0.55, *p* = 0.59, *I^2^_res_* = 47.0%, *τ^2^* = 0.006) or TNF-α (Age: *t* = -0.81, *p* = 0.44, *I^2^_res_* = 42.7%, *τ^2^* = 0.012; BMI: *t* = 0.72, *p* = 0.52, *I^2^_res_* = 56.1%, *τ^2^* = 0.012; Gender: *t* = 0.4, *p* = 0.70, *I^2^_res_* = 47.7%, *τ^2^* = 0.015), although they all showed substantial heterogeneity.

## Sensitivity analysis

Two studies were included in the main analysis for CRP that only assessed trauma in the form of parental absence, yet when these were excluded from the analysis, effect sizes remained significant (*z* = 0.09, *df* = 15, *p* = 0.001, 95%-*CI* = 0.04 - 0.14) at similar levels of heterogeneity (*p* < 0.001, *I^2^* = 65.7%, *τ^2^* = 0.005). When we excluded studies that exclusively considered clinical participants, who may putatively show exacerbated levels of inflammatory markers, effect sizes and heterogeneity for CRP (*z* = 0.09, *df* = 15, *p* < 0.001, 95%-*CI* = 0.05 - 0.13, *I^2^* = 74.4%, *τ^2^* = 0.004), IL-6 (*z* = 0.07, df = 12, *p* = 0.01, 95%-*CI* = 0.02 - 0.13, *I^2^* = 49.5%, *τ^2^* = 0.004) and TNF-α (*z* = 0.25, *df* = 7, *p* < 0.001, 95%-*CI* = 0.15 - 0.35, *I^2^* = 42.1%, *τ^2^* = 0.08) showed only minimal deviation. When studies with prospective assessment of childhood trauma were considered separately from studies with retrospective assessment of childhood trauma, prospective studies showed greater effect sizes but similar heterogeneity (*z* = 0.14, *df* = 2, *p* < 0.001, 95%-*CI* = 0.08 - 0.20, *I^2^* = 75.0%, *τ^2^* = 0.002) that retrospective studies (*z* = 0.08, *df* = 14, *p* = 0.002, 95%-*CI* = 0.03 - 0.13, *I^2^* = 61.3%, *τ^2^* = 0.004). This analysis was possible for CRP only. To assess whether different measures of trauma were associated with differences in effect sizes and heterogeneity, subgroup analyses for individual measures were conducted, although this was possible for CRP only. CTQ was associated with the greatest effect sizes (z = 0.16, df = 5, p = 0.002, 95%-CI = 0.06 - 0.26, I^2^ = 31.9%, τ^2^ = 0.005), compared with non-standardised measures (z = 0.09, df = 4, p = 0.002, 95%-CI = 0.03 - 0.15, I^2^ = 83.7%, τ^2^ = 0.004) and the RFQ, which did not reach significance (z = 0.05, df = 2, p = 0.21, 95%-CI = -0.03 - 0.13, I^2^ = 63.2%, τ^2^ = 0.003). As the RFQ does not assess childhood sexual abuse, we excluded studies using the RFQ from the analyses for CRP and IL-6, however this was not associated with considerable changes in effects sizes or heterogeneity for either CRP (z = 0.11, df = 14, p < 0.001, 95%-CI = 0.06 - 0.15, I^2^ = 70.1%, τ^2^ = 0.004) or IL-6 (z = 0.08, df = 13, p = 0.008, 95%-CI = 0.02 - 0.14, I^2^ = 45.6%, τ^2^ = 0.005).

## Publication bias

Visual inspection of funnel plots suggested that publication biases were unlikely; in line with this, both Egger’s and Begg’s test provided no evidence for publication bias for either CRP (Egger’s *p* = 0.66, Begg’s *p* = 0.49), IL-6 (Egger’s *p* = 0.12, Begg’s *p* = 0.73) or TNF-α (Egger’s *p* = 0.65, Begg’s *p* = 0.65). However, meta-regression for selection bias scores showed significance for IL-6 (*t* = 3.6, *p* = 0.003, *I^2^_res_* = 0.0%, *τ^2^* = 0.0) and a similar trend for CRP (*t* = 1.84, *p* = 0.08, *I^2^_res_* = 67.7%, *τ^2^* = 0.005), but not TNF-α (*t* = 0.26, *p* = 0.79, *I^2^_res_* = 43.8%, *τ^2^* = 0.01).
